# Alleviation of cadmium toxicity in *Zea ma*ys L. through up-regulation of growth, antioxidant defense system and organic osmolytes under calcium supplementation

**DOI:** 10.1371/journal.pone.0269162

**Published:** 2022-06-22

**Authors:** Muhammad Kaleem, Farah Shabir, Iqbal Hussain, Mansoor Hameed, Muhammad Sajid Aqeel Ahmad, Anam Mehmood, Waseem Ashfaq, Saima Riaz, Zarbakht Afzaal, Muhammad Faisal Maqsood, Ummar Iqbal, Syed Mohsan Raza Shah, Muhammad Irshad

**Affiliations:** 1 Department of Botany, University of Agriculture, Faisalabad, Pakistan; 2 Department of Botany, Government College University, Faisalabad, Pakistan Department of Botany, Government Associate College for Women Layyah, Layyah, Pakistan; 3 Department of Botany, Government Associate College for Women Layyah, Layyah, Pakistan; 4 Department of Bioinformatics & Biotechnology, Government College University, Faisalabad, Pakistan; 5 Department of Plant Breeding and Genetics, University of Agriculture, Faisalabad, Pakistan; Zagazig University, EGYPT

## Abstract

Calcium (Ca) is a macronutrient and works as a modulator to mitigate oxidative stress induced by heavy metals. In this study, we investigated the role of Ca to ameliorate the Cd toxicity in Zea mays L. by modulating the growth, physio-biochemical traits, and cellular antioxidant defense system. Maize genotype Sahiwal-2002 was grown under a controlled glasshouse environment with a day/night temperature of 24 ± 4°C/14 ± 2°C in a complete randomized design with three replications and two Cd levels as (0 and 150 μM) and six regimes of Ca (0, 0.5, 1, 2.5, 5, and 10 mM). Maize seedlings exposed to Cd at 150 μM concentration showed a notable decrease in growth, biomass, anthocyanins, chlorophylls, and antioxidant enzymes activities. A higher level of Cd (150 μM) also caused an upsurge in oxidative damage observed as higher electrolyte leakage (increased membrane permeability), H_2_O_2_ production, and MDA accumulation. Supplementation of Ca notably improved growth traits, photosynthetic pigments, cellular antioxidants (APX, POD, and ascorbic acid), anthocyanins, and levels of osmolytes. The significant improvement in the osmolytes (proteins and amino acids), and enzymatic antioxidative defense system enhanced the membrane stability and mitigated the damaging effects of Cd. The present results concluded that exogenously applied Ca potentially improve growth by regulating antioxidants and enabling maize plants to withstand the Cd toxicity.

## Introduction

While growing in natural environments, plants are exposed to various environmental stresses that limit yield and productivity [1]. Heavy metal pollution is spreading in cultivated lands and is causing severe environmental hazards to crop plants, human health and ecosystems [2]. Cadmium (Cd) is regarded as the most toxic heavy metal, typically when present in agricultural lands due to its higher mobility and toxicity [3]. Plants can readily absorb Cd directly through roots from the soil along with essential nutrients [4]. Like other heavy metals, Cd causes structural changes in plants and adversely affects the morphological, physiological, and biochemical mechanisms eventually leading to loss of agricultural productivity [5]. Cadmium is highly toxic to plants and imposes negative influences on growth and entire metabolism [6]. It is typically non-essential for agricultural crops as no known role is ascribed to Cd in the growth and development of crop plants [1]. Therefore, Cd even in minor concentrations disturbs photosynthesis, changes the ultrastructure of the chloroplast, increases lipid peroxidation and enhance the production of ROS that leads to oxidative damage [7–9]. The dynamics of Cd in the rhizosphere depends on uptake mechanisms, translocation, and toxicity of Cd in plants. In crop plants, the toxicity of Cd reduces uptake and translocation of nutrients and water, increases oxidative damage, disrupts plant metabolism, and inhibits plant morphology and physiology [10]. In wheat, for example, Cd exposure reduced plant growth, yield, photosynthetic efficiency, hormones, proteins and increased MDA, H_2_O_2_, soluble sugars and prolines [11]. Another direct effect of high Cd is the production of excessive ROS (H_2_O_2_, OH^−^, O_2_^.-^_,_^1^O_2_) resulting in lipids peroxidation which ultimately reduces plant growth [12].

Many defensive mechanisms are induced in plants to counteract Cd toxicity mainly by hyper production of antioxidants (non-enzymatic or enzymatic) to control heavily produced ROS [5, 13]. These enzymatic antioxidants (like peroxidase, superoxide dismutase, ascorbate peroxidase, and catalases), and non-enzymatic antioxidants (such as α-tocopherol and glutathione) have been reported to successfully mitigate Cd-induced oxidative damage in many crop plants [12, 14]. Those plants protected by antioxidants show improved growth and yield [15]. Other reports show that heavy metals may result in hyper-accumulation of proteins as an effective strategy to mitigate Cd-induced toxicity [16]. The supplementation of Ca strengthens the anti-oxidants, reduction in lipid peroxidation, and increases proline accumulation and synthesis, clearly indicating protection against Cd stress by increasing the maintenance of systematic resistance criteria [17].

Calcium (Ca^2+^) is an essential macromolecule and divalent cation that performs an imperative role in membrane permeability, metabolism and signal transduction [16, 18, 19]. It is a central regulator in the physio-chemical process and regulates plant growth [20]. Exogenously applied Ca alleviates oxidative stress by chelating with target proteins (for instance calcium-binding proteins) and activating the antioxidant enzymes [21, 22]. Though, the induction of the antioxidative defense system by Ca is not yet elucidated sufficiently, some reports support that Ca is involved in the modulation of genes for antioxidant enzymes [23]. Ca mitigates Cd toxicity in plants by modifications in the morphological and physiological processes [20, 24]. For example, Ca maintains permeability of membranes by a reduction in peroxidation of lipids and solute leakage which ultimately reduces oxidative stress caused by Cd stress [25]. Calcium is involved in controlling basic functions such as photomorphogenesis, cell division, cell elongation, stress responses, and the maintenance of membrane structure and functions [24, 26]. In other reports, Ca improved growth and photosynthesis by restricting Cd translocation and accumulation, scavenging ROS, enhancing antioxidant levels, and maintaining Ca-dependent signal transduction [27, 28]. Still, the ameliorative role of Ca to alleviate heavy metals toxicity remains inconclusive and therefore it is imperative to investigate its specific roles and associated mechanisms in improving the growth of *Zea mays* L. seedlings.

Maize is a valuable cereal crop and provides food for humans as well as fodder for livestock. It contributes to 36% (782 Mt) of global grain production [29]. Maize seeds are enriched with energy as 100g seeds contain 365 kilocalories of energy [30]. Among worldwide production, 70–80% of maize is used as food and was ranked third in Pakistan for consumption after wheat and rice. Pakistan was ranked 18^th^ in the production with 6130 thousand tons of maize produced annually that was cultivated at 1334 thousand hectares [31]. The requirement for maize production has significantly increased recently due to excessive usage in the wet milling industry as well as food for poultry [32]. This needs not only to increase the cultivation area but also the exploration of new promising techniques to increase crop survival and yield under stressful environments like in soils contaminated with heavy metals [33]. Maize plant is tolerant to certain levels of Cd, however, when exposure to high levels causes negative effects on different growth stages that are more severe on the emergence of the seedlings and at fourth leaf stage [34].

Cadmium contamination is gradually increasing in soils and is causing significant crop losses. Therefore, there is a dire need to devise new strategies to combat the problems associated with Cd toxicity. Considering many critical roles played by Ca in plant growth and metabolism, it was hypothesized that Ca supplementation should effectively ameliorate the adverse effects of Cd imposed on germinating seeds of maize. The research questions included probing into the toxic effects of Cd on the growth, physio-biochemical characteristics and to what extent supplemental Ca can alleviate Cd toxicity in maize. Since the information is lacking regarding the mechanisms involved in the amelioration of cadmium toxicity, this work will suggest future directions to work out the underlying molecular mechanisms involved in the mitigation of heavy metals in different plants.

## Materials and methods

### Plant materials

Maize seeds (Sahiwal-2002) were obtained from Maize and Millets Research Institute (MMRI), Yousaf Wala Sahiwal, Pakistan. Seeds were immersed in 30% (v/v) H_2_O_2_ for 5 min. for sterilization, washed with deionized water for 24 h, dried and stored till experimentation.

### Selection of cadmium and calcium levels

A preliminary experiment was conducted with different concentrations of Cd in form of cadmium chloride (0, 50, 100, 150, 200 μM), and Ca in form of calcium nitrate (0, 0.5, 1, 2.5, 5 and 10 mM) were used to screen the optimal levels of Cd and Ca. Based on preliminary experiment’s results, the 150 μM Cd stress caused 50% growth and germination inhibition, while 5 and 10 mM Ca showed best results to improve the negative impacts of Cd.

### Treatment application and experiment layout

Seeds were placed in Petri dishes lined with a double layer of Whatman # 02 filter papers. The surface of each filter paper was moistened with 15 mL of H_2_O and kept in dark condition 25 ± 2°C for 48 h. After germinations, six seeds were planted in plastic pots (depth; 40 cm and diameter; 35 cm) in sterilized sand with a particular particle size of 0.25 mm. The sand was soaked for 24 h in 30% (v/v) HCl solution to remove all cations and anions and then thoroughly rinsed with deionized water three times (with 24 h soaking). All pots were arranged as a CRD design with 3 replications under a controlled glasshouse environment with a day/night temperature of 24 ± 4°C/14 ± 2°C and of relative humidity 58–60% [35]. The ½-strength Hoagland’s (Hoagland 1938) nutrient solution was applied to all pots by saturating the sand at an interval of 2 days until the complete emergence of the seedlings (20 days). Once a week, the solution was completely drained by applying enough Hoagland’s nutrient solution to ensure replacing any existing solution left in sand. After complete emergence, seedlings were thinned to 4 plants in each pot. The seedlings were then treated either with Cd^2+^ (using CdCl_2_) or Ca (using Ca(NO_3_)_2_) by using analytical grades prepared in Hoagland solution. The pH of the sand medium (6.7) and nutrient solution (7.5) was adjusted with HCl or NaOH and periodically measured with a portable pH meter (Ino LAB pH/Cond 720, WTW series).

### Plant sampling and measurements

Plants materials were sampled at the seedling stage (4–6 leaf stage; 30 days after seedlings emergence) to determine plant growth attributes, physio-biochemical traits, ROS, and enzymes of an antioxidants defense system. Transplants were washed with distilled water and growth attributes were recorded. Leaf samples of maize seedlings were frozen at –80°C for physio-biochemical traits and antioxidants. Sampled seedlings were dried in an oven at 70°C to achieve a constant dry weight for determination of root (RDW) and shoot (SDW) dry weight.

### Growth parameters

The shoot length (SL) of plants from each treatment was measured from sand level to the topmost leaf of the plant. The roots of seedlings were carefully removed from the sand for recording root length (RL). Root (RFW) and shoot fresh weight (SFW) of seedlings were measured immediately after excision. The leaf area (LA) was estimated by measuring length and width according to Kaleem and Hameed [36]

Leafarea(cm2)=maximumlength×maximumwidth×correctionfactor0.68)

### Photosynthetic pigments measurement

Chlorophyll contents were assessed as described by Arnon [37] and carotenoids following the method of Davis [38]. For the appraisal of chlorophyll contents, 0.1 g of leaf sample was grounded in 5 mL of acetone (80%). The extract was filtered through a Whatman # 02 filter paper (GE Healthcare, UK) and absorbance was recorded through a spectrophotometer (Hitachi U-2910, Tokyo, Japan) at 645, 663, and 480 nm. The values of photosynthetic pigments were calculated by using the following formulas.

Chl. *a* (mg/g of leaf fresh weight) = [12.7(OD663)-2.69(OD645)] x V/1000 x W

Chl. *b* (mg/g of leaf fresh weight) = [22.9(OD645) - 4.68(OD663)] x V/1000 x W

Total Chl. (mg/g of leaf fresh weight) = [20.2(OD645) + 8.02 (OD663)] x V/1000 x W

Carotenoids (g/ ml of fresh leaf) = {[(OD480) +0.114 (OD663)– 0.638 (OD645)]/2500}

Where V characterizes the volume of acetone and (FW) showed the leaf fresh weight.

### Determination of relative membrane permeability

The fresh leaf samples were collected and washed thoroughly with 4 changes of water to eradicate any adhered electrolytes on the surface. The leaves were cut into small discs with a borer and placed in the small glass test tube containing deionized water (10 mL), The EC_o_ was measured with the help of Cond/Salinity meter (TPS AQUA-CPA). The test tubes were incubated for 24 h at 4°C and EC_1_ was measured. The tubes were then wrapped with aluminium foil, autoclaved for 10 min. at 100 kPa and EC_2_ was recorded. The ratio of % ion leakage was computed as designated by Yang et al. [39].


RMP(%)=(EC1‐EC0)/(EC2‐EC0)×100

Where, RMP: relative membrane permeability, EC0; Electrical conductivity before incubation, EC1; Electrical conductivity after incubation, EC2; Electrical conductivity after autoclave.

### Anthocyanin contents measurement

Anthocyanin content was appraised according to the method of Giusti & Wrolstad [40]. The 0.1 g of a leaf was pulverized in trichloroacetic acid (TCA) by using a pestle and mortar. The homogenized material was transferred to test tubes and shifted to a water bath at 80°C for 20 min. Homogenized material was centrifuged at 12,000 *xg* for 10 min. in the absorbance was noted at 516 and 700 nm using a spectrophotometer (Hitachi U-2910, Tokyo, Japan). Acetone was run as blank and the amount of monomeric anthocyanin contents was calculated as follows.


Monomericanthocyaninpigments(mg/L)=(A×MW×DF×1000)/(ε×1)

Where, A = (A510-A700), MW = 449.2 and ε = 26900 [ε is the molar absorptivity measured the amount of cyanidin-3-glucoside pigment and DF is the dilution factor].

### Oxidative stress markers (MDA and H_2_O_2_)

Lipids peroxidation (LPX) was quantified by means of malondialdehyde (MDA contents) according to the method of Heath and Packer [41]. LPX content was determined by the reaction of thiobarbituric acid-TBA with MDA. The 0.25 g leaf sample was grinded in 500 μL of TCA (0.1%) and then centrifuged at 15,000 *xg*. An aliquot (1 mL) was taken and mixed with 2 mL of 0.5% of TBA and 20% TCA. Test tubes containing reactants were incubated at 85°C for 20 min. and reaction was terminated in an icebox. Absorption was recorded at 532 and 600 nm by spectrophotometer (Hitachi U2910, Tokyo, Japan). All absorption ODs (at 532nm) were subtracted from 600 nm. LPX concentration was calculated by using 155 mM cm^-1^ as an extinction coefficient.

The Amount of H_2_O_2_ was quantified by measuring the oxidation of ferrous ions medicated by peroxidase and ferric ions react with the xylenol [42]. Leaf sample 0.5 g was grounded in 5 mL of 10 mM sodium phosphate buffer (SPB). Centrifugation of homogenized material was done at 15,000 *xg*. A 2 mL of aliquot was reacted with the assay reagent containing 200 mM sorbitol, 200 μM xylenol, 50 mm H_2_SO_4,_ and 500 μ*M* ammonium ferrous sulphate. The reactant material was incubated at 24°C for 1 h and absorption of yellow colour intensity of supernatant was recorded at 560 nm by using a spectrophotometer (Hitachi U-2910, Tokyo, Japan). The final concentration of H_2_O_2_ was calculated by using the coefficient of emission (0.28 mmol^–1^ cm^–1^).

### Cellular antioxidants (APX and POD)

The maize seedlings, leaves were grounded in liquid nitrogen and extracted with 1 mM L^–1^ of 5% polyvinylpyrrolidone, and, 50 mM sodium phosphate buffer (SPB) having 1.0% w/v at pH 7.8 as homogenizing material. The extracted material was centrifuged at 15,000 *xg*. Enzyme crude extract was stored at 4°C for 36 h until analysis.

### Ascorbate peroxidase activity (APX)

Activity of APX was quantified by oxidation of ascorbate [43]. The reaction was started by adding 10 μL of crude enzyme extract to 2 mL of assay reagent (30% H_2_O_2,_ 0.5 mM C_6_H_8_O_6_, and sodium phosphate buffer (SPB) having pH 7.2,). After 30 s of reaction initiation, a shift in absorption was noted at 290 nm for 4 min. on a spectrophotometer (Hitachi U-2910, Tokyo, Japan). Activity of enzyme was estimated through extinction coefficient (2.8 mM cm^-1^), while the specific activity of the enzyme was calculated on the basis of protein contents and expressed as an mg^–1^ min.^-1^ FW.

### Peroxidase activity (POD)

Peroxidase activity was appraised spectrophotometrically by using the method of Goliber [44] based on oxidisation of guaiacol in the presence of H_2_O_2_ and expressed as a Units mg^-1^ proteins. A 20 μL of the enzyme extract was added to the assay reagent (20 mM guaiacol, 10 mM H_2_O_2,_ and 0.1 M phosphate buffer) and volume was maintained up to 3 mL. Enzyme activity was measured at 460 nm after 60 s interval through a spectrophotometer (Hitachi U-2910, Tokyo, Japan). Enzyme specific activity was expressed on the base of proteins.

### Ascorbic acid determination

Ascorbic acid was determined as described by Nino and Shah [45]. Plant tissues (100 mg) were pulverized in thiobarbituric acid (TBA) and centrifuged 10,000 *×g* for 10 min. An aliquot (500 μL) was taken with 500 μL of dithiocarbamate (DTC) in glass tubes. Reactants were left for ½ h at 37°C. Test tubes containing reactant material was transferred to the ice bath to terminate the reaction. After that, 2 mL of dilute H_2_SO_4_ was mixed slowly and leftover for ½ h at 37° C in an incubator. The extracted material was centrifuged at 12,000 *xg*. The shift in absorption was measured at 520 nm with the help of a spectrophotometer (Hitachi U-2910, Tokyo, Japan).

### Total free amino acids

The free amino acid was quantified followed by Hamilton & Van-Slyke [46] method. The 0.1 g of the leaf sample was grinded and immersed in a potassium phosphate buffer (SPB) overnight. After incubation, 1 mL of plant extract was transferred to 25 mL test tubes after adding 1 mL each of 10% ninhydrin and 2% of pyridine solution. The test tubes containing reactants were placed in a boiling water bath for 1 h. The final volume of samples was made to 25 mL by using deionized H_2_O. Absorbance was recorded at 570 nm spectrophotometrically (Hitachi U-2910, Tokyo, Japan) and resulting absorbance was compared with the standard curve plotted for leucine.

### Soluble proteins

Soluble proteins were appraised following Lowry et al. [47]. Plant sample (0.1 g) was grounded in 50 mM sodium phosphate buffer (SPB) having pH 6.8. The extracted aliquot (500 μL) was mixed in 0.3 mL of deionized H_2_O and 3 mL of Bio-Rad protein assay dye and vortexed for 15 s. The absorbance was measured spectrophotometrically at 750 nm (Hitachi U-2910, Tokyo, Japan). Soluble proteins were estimated by comparing the absorbance of samples with bovine serum albumin (BSA) using a standard value.

### Statistical analysis

Statistical analysis and data visualization were executed by using R statistical software (R Core Team, 2021) through the R integrated development environment in R Studio (R Studio Team, 2021). Data within three replicates were subjected to an analysis of variance (ANOVA) and means values were compared by using the Tukey pairwise comparison test at (*P≤* 0.05) to test the effects of Ca under Cd stress on maize seedlings. Bar plots were constructed by using the “agricolae” package of the R software. The effect of Ca and Cd treatments was assessed by using multivariate analysis (PCA by ggbiplot), correlation matrix (ggbiplot2) and heatmaps were plotted by customized code (pheatmap) by using R statistical software (R Studio Team, 2021). Response curves under cadmium and calcium stress treatments were constructed by fitting a generalized linear model (GLM) in CONACO version 5 for windows.

## Results

### Plant growth traits

Growth traits such as SL, RL, SFW, SDW, RFW, RDW and LA significantly (P ≤ 0.05) decreased at Cd applied at 150 μm concentration as compared to non-stressed plants (0 μ*M*). The reduction was 75.3%, 88.3%, 77.83%, 98.6%, 91.6%, 99.86%, 68.1%, respectively. However, different levels of Ca significantly alleviated Cd toxicity and enhanced all growth traits. The increase in growth traits was more obvious in response to a higher level of Ca applied at 10 m*M* under Cd stress (150 μm). The percent increase was 64.6%, 28.4%, 66.2%, 23.2%, 40.3%, 46.4%, and 35.5, respectively (Table 1).

**Table 1:** Morphological characteristics of maize seedlings under Ca and Cd treatments.

Cd stress (μM)	Ca treatments (mM)	SL (cm)	RL (cm)	SFW (g)	SDW (g)	RFW (g)	RDW (g)	LA (cm^2^)
**0**	0	57.44±5.71^d^	18.66±1.32^d^	32.65±1.66^d^	3.12±0.13^d^	2.43±0.08^d^	0.26±0.02^c^	49.51±2.23^d^
	0.5	66.22±1.92^c^	23.61±1.28^d^	40.72±1.82^c^	3.55±0.09^d^	3.09±0.16^c^	0.36±0.04^c^	57.79±0.86^c^
	1	66.22±1.34^c^	30.94±0.80^c^	47.14±0.95^c^	4.46±0.15^c^	3.29±0.04^c^	0.45±0.03^b^	60.99±4.06^c^
	2.5	73.00±0.84^b^	40.66±2.47^b^	48.31±1.85^c^	4.54±0.36^c^	3.73±0.06^b^	0.56±0.02^b^	73.04±3.07^b^
	5	93.66±2.95^b^	45.61±3.11^b^	59.00±1.51^b^	5.87±0.22^b^	4.63±0.12^a^	0.62±0.02^a^	75.91±3.60^b^
	10	105.7±2.01^a^	65.94±2.84^a^	81.91±3.19^a^	7.75±0.10^a^	5.60±0.11^a^	0.66±0.03^a^	95.87±2.34^a^
**150**	0	24.64±2.38^d^	11.61±2.22^d^	22.17±1.74^d^	1.40±0.22^c^	1.35±0.17^c^	0.14±0.02^c^	31.96±1.06^d^
	0.5	52.44±3.75^c^	18.95±0.80^c^	28.45±1.63^d^	1.61±0.03^c^	1.99±0.11^c^	0.16±0.01^c^	39.30±0.94^d^
	1	63.22±3.74^b^	20.34±2.35^c^	56.50±2.39^c^	1.91±0.39^c^	2.11±0.15^b^	0.21±0.01^b^	53.40±5.49^c^
	2.5	72.44±2.92^b^	30.60±1.45^b^	73.11±2.11^b^	2.81±0.28^b^	2.94±0.26^a^	0.23±0.01^b^	54.09±5.11^c^
	5	82.77±4.29^a^	35.10±2.69^b^	77.96±5.19^b^	3.26±0.11^b^	3.10±0.18^a^	0.32±0.03^a^	62.96±1.46^b^
	10	91.55±3.83^a^	51.14±3.16^a^	90.64±1.52^a^	4.97±0.20^a^	3.11±0.09^a^	0.30±0.04^a^	76.99±2.57^a^

Means provided with error bars; in columns different letter indicates significance (P≤0.05) between treatments

**Abbreviation:** Shoot length **(SL)**; Root length **(RL)**; Shoot fresh weight **(SFW)**; Shoot dry weight **(SDW)**; Root fresh weight **(RFW)**; Root dry weight **(RDW)**; Leaf area **(LA)**

### Photosynthetic pigments

Under Cd stress (150 μM), a significant (*P* ≤ 0.05) reduction occurred in the concentration of photosynthetic pigments such as Chl *a*, Chl *b*, carotenoids (Caro), and total chlorophyll (T.Chl) of maize seedlings. The reduction was 96.4%, 98.6%, 99.8%, and 94.9% as compared to the non-stressed control (Ca-0 mM) and stressed (Cd-0 μM) seedlings of the maize. The Exogenously supplied Ca significantly increased photosynthetic pigments (Chl *a*, Chl *b*, Caro, T.Chl) both in Cd stressed and non-stressed seedlings. Calcium applied at 10 mM level was more beneficial in increasing chlorophyll and carotenoids contents of maize seedlings at 150 μM concentration of Cd. The percent increase was 286.2%, 266.0%, 215.4% and 140.8%, respectively (Table 2).

**Table 2:** Physiological traits of maize seedlings under various levels of Ca and Cd treatments.

Cd stress (μM)	Ca levels (mM)	Chl *a* (mg g^-1^ FW)	Chl *b* (mg g^-1^ FW)	Caro. (mg g^-1^ FW)	T. Chl (mg g^-1^ FW)	APX (Units mg^-1^ Pro)	POD (Units mg^-1^ Pro)
**0**	0	9.76±0.25^c^	2.34±0.09^c^	0.35±0.01^d^	10.69±0.26^c^	0.92±0.05^c^	0.55±0.11^c^
	0.5	12.98±0.54^c^	2.84±0.23^c^	0.43±0.01^c^	11.43±0.75^c^	1.02±0.04^c^	0.60±0.12^c^
	1	16.56±0.84^b^	4.44±0.15^b^	0.48±0.02^c^	14.02±0.64^b^	1.98±0.02^b^	0.73±0.04^b^
	2.5	18.32±0.62^a^	4.57±0.19^b^	0.51±0.02^b^	14.93±0.83^b^	1.93±0.02^b^	0.78±0.03^b^
	5	18.60±0.38^a^	5.48±0.18^a^	0.52±0.03^b^	16.00±1.06^a^	1.98±0.06^b^	0.87±0.07^a^
	10	19.26±0.76^a^	6.49±0.10^a^	0.70±0.02^a^	16.02±0.25^a^	2.17±0.03^a^	0.91±0.06^a^
**150**	0	3.63±0.22^d^	1.41±0.03^c^	0.13±0.01^c^	5.02±0.23^d^	1.27±0.03^c^	0.45±0.09^d^
	0.5	5.33±0.19^c^	2.85±0.12^c^	0.17±0.01^c^	6.35±0.46^c^	1.39±0.19^b^	0.72±0.02^c^
	1	6.50±0.82^c^	3.08±0.08^b^	0.22±0.01^b^	7.25±0.45^c^	2.01±0.04^b^	0.85±0.06^b^
	2.5	10.72±0.47^b^	3.47±0.14^b^	0.27±0.01^b^	8.22±0.22^b^	2.17±0.03^a^	0.82±0.06^b^
	5	12.42±1.11^b^	4.13±0.13^a^	0.32±0.03^a^	9.18±0.37^b^	2.10±0.02^a^	1.10±0.01^a^
	10	14.02±0.53^a^	5.16±0.20^a^	0.41±0.03^a^	12.09±0.52^a^	2.22±0.01^a^	1.12±0.06^a^

Means provided with error bars; in columns different letter indicates significance (P≤0.05) between treatments

**Abbreviations:** Chlorophyll *a* **(Chl *a*)**; Chlorophyll *b* **(Chl *b*)**; Carotenoids **(Caro)**; Total chlorophyll **(T. Chl)**; Ascorbate per oxidase **(APX)**; Peroxidase **(POD)**; Protein **(Pro)**

### Antioxidative enzyme activities

Mean values for antioxidant activity was higher in Cd stressed (150 μM) as compared to non-stressed plants (0 μM). However, the activity of APX significantly enhanced as levels of Ca increased both in non-stressed (0 μM) and stressed plants (150 μM). The maximum activity of the APX (74.8%) and POD (148.9%) was recorded at 10 mM Ca concentration as compared to its control. However, significant reduction was recorded in stressed seedlings (150 μM) as compared to non-stressed (0 μM) control plants under no supplementation of the Ca as 98.8% and 99.5%, respectively (Table 2).

### Anthocyanin and relative membrane permeability

**Fig 1:**
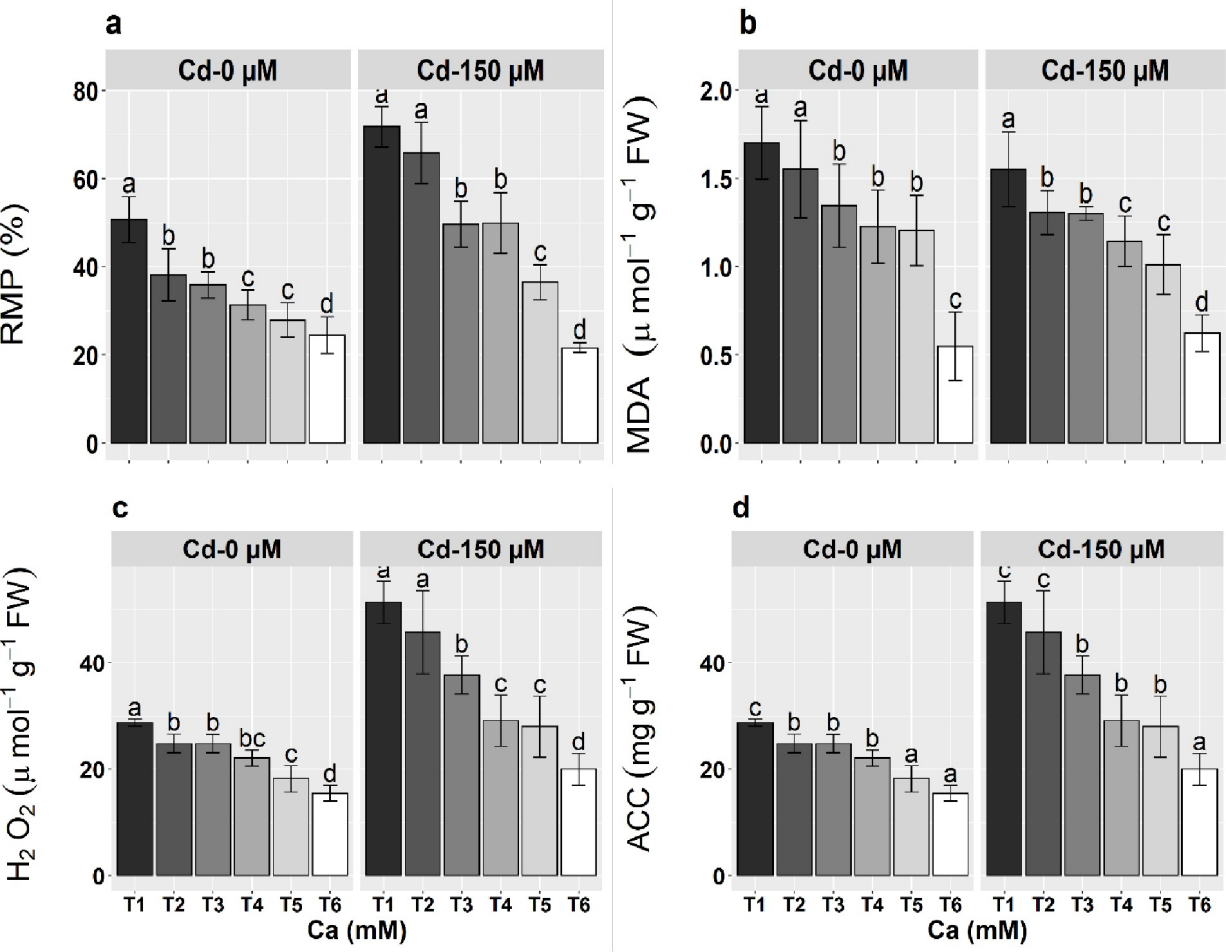
Effect of calcium (Ca; T1-0 m*M*, T2-0.5 m*M*, T3-1 m*M*, T4-2.5 m*M*, T5-5 m*M*, T6-10 m*M*) and cadmium (Cd) treatments on the a) relative membrane permeability (RMP), b) melanoaldehyde contents, c) H_2_O_2_, and d) anthocyanine contents (ACC) of maize seedlings. Means ± SE provided with error bars; different letter indicates significance (*P≤*0.05) between Ca and Cd treatments.

Under Cd stress, maximum RMP values were observed which indicate a high level of electrolyte leakage due to membrane damage. A significant (P ≤ 0.05) reduction (69.9%) was observed as the level of Ca increased (Fig 1A). Cadmium applied at 150 μm level and without any Ca supplementation had the most toxic effects as the highest electrolyte leakage was observed at this treatment level. Anthocyanin contents (ACC) under both treatments of Cd significantly increased as levels of Ca increased. Maximum increase (133.4%) in ACC were noticed in stressed plants (150 μM) at 10 mM Ca concentration (Fig 1D).

### Lipid peroxidation (LPX) and ROS

The accumulation of H_2_O_2_ and MDA significantly increased in maize seedlings under Cd stress. However, the elevated levels of Ca significantly reduced the generation of H_2_O_2_ and LPX. The LPX in terms of MDA contents significantly decreased as the level of Ca increased in the growth medium of the stressed seedlings. The maximum decrease (59.9%) was observed under 10 mM concentration of Ca. The 10 mM concentration of Ca also cause reduction (61.2%) in the generations of H_2_O_2_ in Cd stressed seedlings (Fig 1C).

### Organic osmolytes

**Fig 2:**
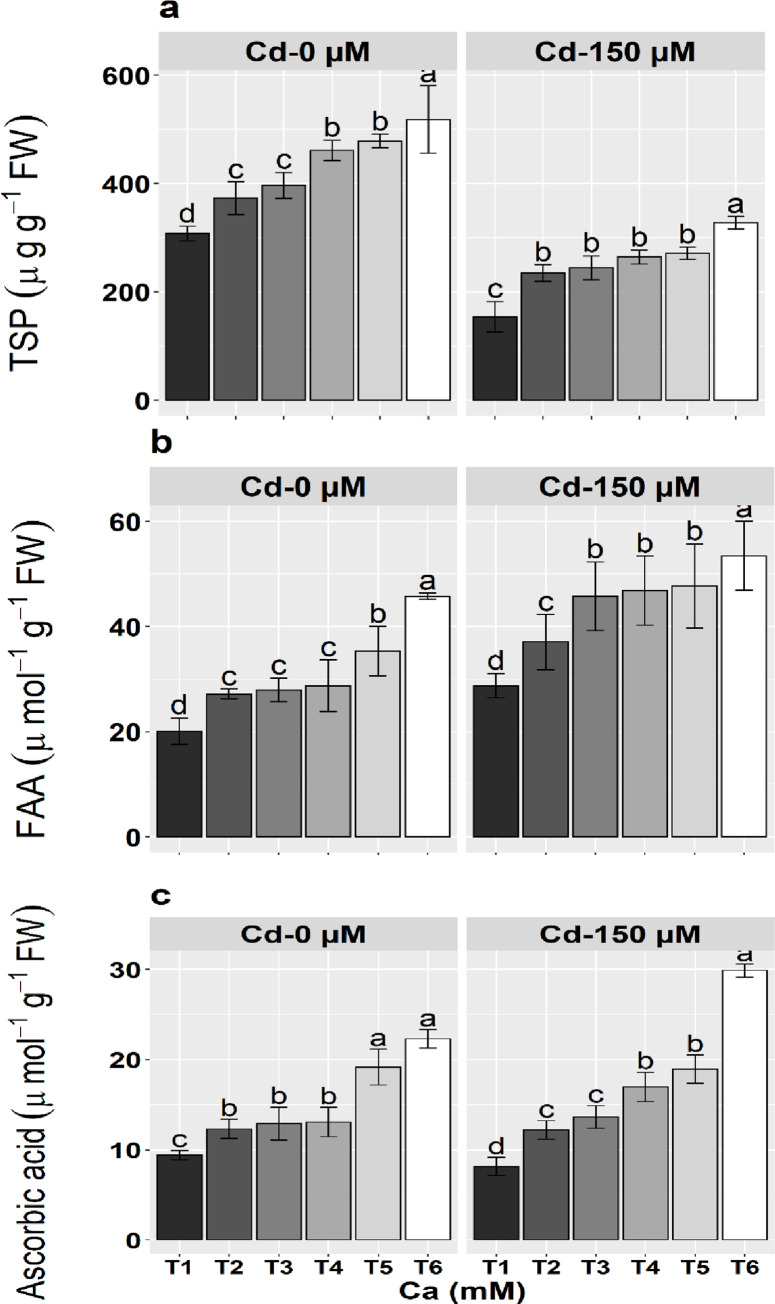
Effect of calcium (Ca; T1-0 m*M*, T2-0.5 m*M*, T3-1 m *M*, T4-2.5 m *M*, T5-5 m *M*, T6-10 m*M*) and cadmium (Cd) treatments on the a) toatl soluble protiens, b) amino acids and c) ascorbic acid contents of maize seedlings. Means ± SE provided with error bars; different letter indicates significance (*P≤*0.05) between Ca and Cd treatments.

Organic osmolyte (proteins and amino acids) production was significantly increased in maize seedlings in stressed and non-stressed maize seedlings. In stressed plants, the increase was 133.2% and 66.6%, respectively. However, soluble proteins were significantly higher in non-stressed maize seedlings as the level of Ca increased. In Cd stressed seedlings (150 μM), the concentration of soluble proteins significantly increased and the maximum was observed under 10 mM Ca concentration (Fig 2A). Applications of Ca substantially increased the concentration of amino acids in both stressed and non-stressed seedlings and almost parallel results were observed as noted for soluble proteins (Fig 2B).

### Ascorbic acid contents measurement

Ascorbic acid contents were substantially improved as Ca levels increased in maize seedlings under normal and stress conditions. Maximum values of ascorbic contents were observed under 10 m*M* concentration of Ca in both stressed and non-stressed conditions (Fig 2C). The concentration of ascorbic acid was significantly increased (197.0%) in stressed plants at the higher concentration of the Ca in the soil medium.

### Principal component analysis (PCAs)

**Fig 3:**
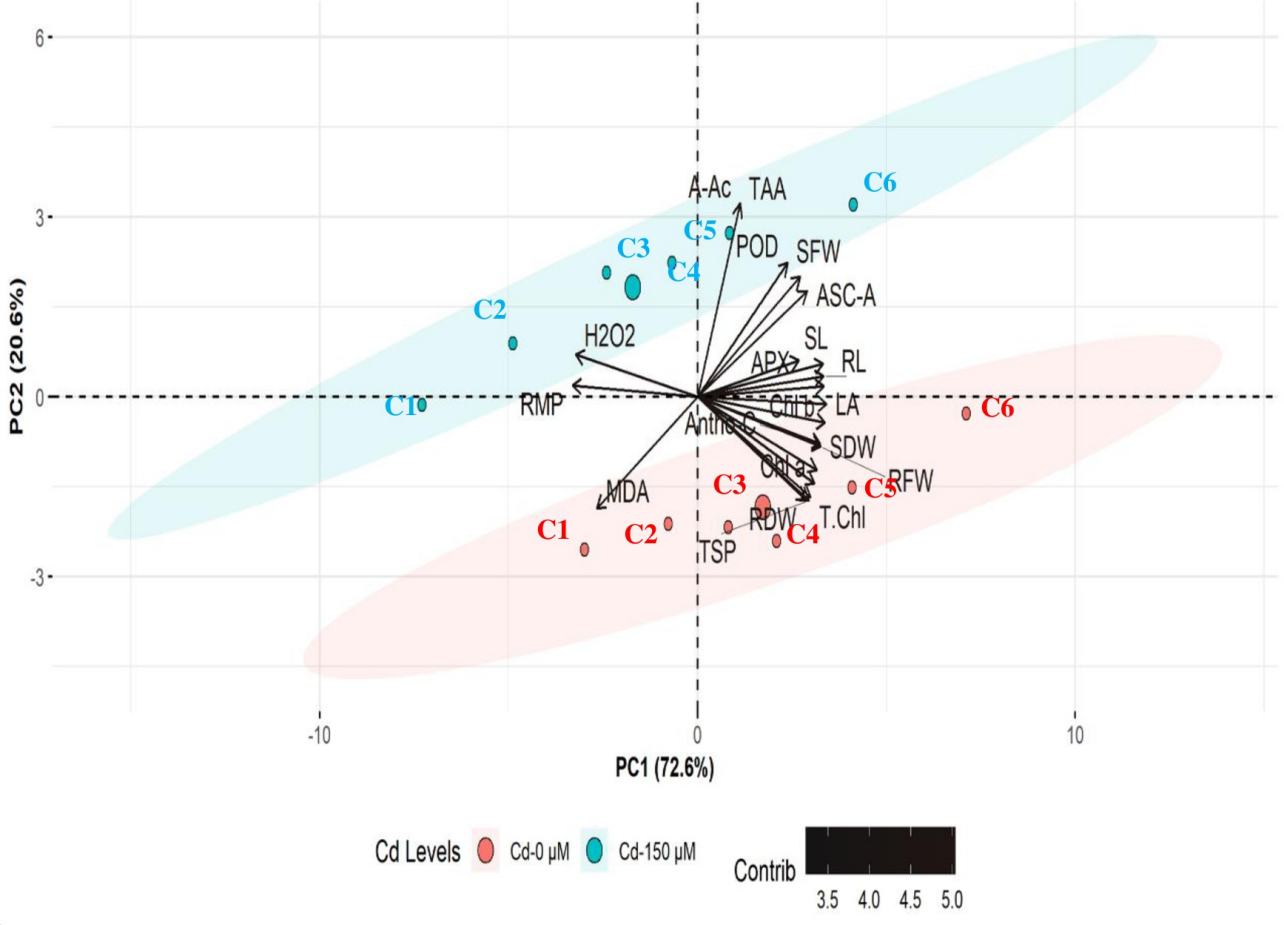
PCA biplot for growth and physio-biochemical traits under Cd and calcium (C1-0 mM, C2-0.5 m*M*, C3-1 m*M*, C4-2.5 m*M*, C5-5 m*M*, C6-10 m*M*) treatments. Abbreviations are given at start of manuscript.

PCAs results demonstrated high variations in the effects of Cd and Ca treatments among different growth and physio-biochemical traits of maize seedlings (Fig 3). The first and second PCAs explained 75.8% and 17.2% (total 93%) variation among treatments and seedlings characteristics. The major contributors to the 150 μM Cd level were amino acids (A-Ac), peroxidase (POD), H_2_O_2_, and RMP with high positive eigenvalues. The activity of antioxidative enzymes (POD, APX, ASC-A), photosynthetic pigments (Chl *a*), and growth traits significantly increased under Cd stress (150 μM) The SFW, TAA, and A-Ac excelled in strong association with a higher concentration of Ca (C5-C6). Under lower levels of Ca *i*.*e*. C1 and C2, the ROS and RMP increased under Cd stress. The major principal components to control plants (0 μM Cd) were RFW, RDW, anthocyanin contents, Chl *b*, T. Chl, carotenoids, TSP, and MDA with negative eigenvalues (Fig 3). Higher concentration of Ca (C6) closely related to growth attributes as LA, SDW, RFW, while C5 contributed to T.Chl, C3 with TSP, C2 and C1 excelled concerning MDA. The variable Antho-C, LA, SDW, RFW, Chl a and T.Chl had shown positive loading of eigenvalues toward the PCA1. and LPX with negative eigenvalues (Fig 3). The Cd stress significantly increased the level of reactive oxygen species, while supplemented Ca significantly increased the antioxidative enzymes activity and growth parameters (Fig 3).

## Correlation matrix

**Fig 4:**
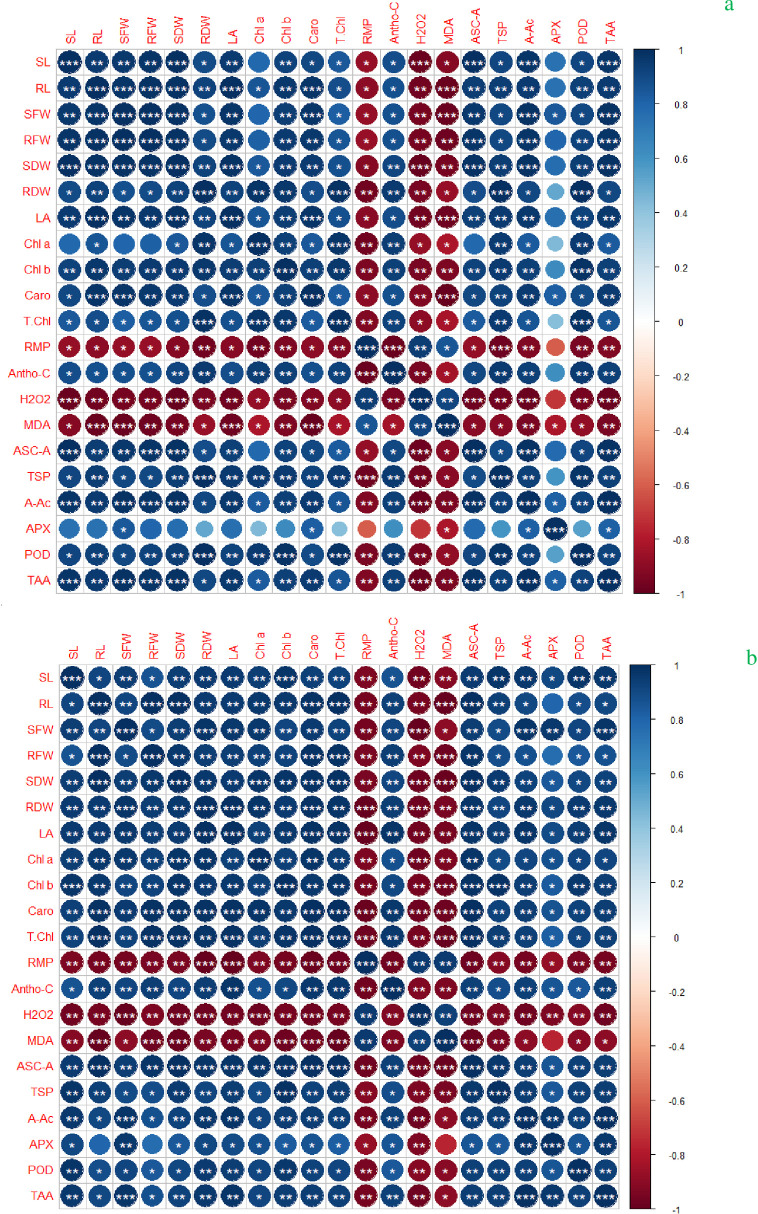
Correlation among morphological and physio-biochemical traits of maize seedlings under (a) under control and (b) Cd stress condition. Abbreviations are given at start of manuscript.

In control plants, anthocyanin contents (Antho-C) was positively correlated with RFW, RDW, SL, LA, Caro, Chl *b* and TSP. The RMP, H2O2, and RMP were negatively correlated with RFW, RDW, Chl *a*, *b*, RL, LA, A-AC and APX (Fig 4A). Under Cd stress, a highly positive correlation was assessed between POD, SFW, and ASC.A, APX, Chl *a*, SL, and RL. However, a strong negative correlation was assessed between H_2_O_2_, RMP, and antioxidant enzymes under Cd-150 μM stress (Fig 4B).

### Clustered heatmap

**Fig 5:**
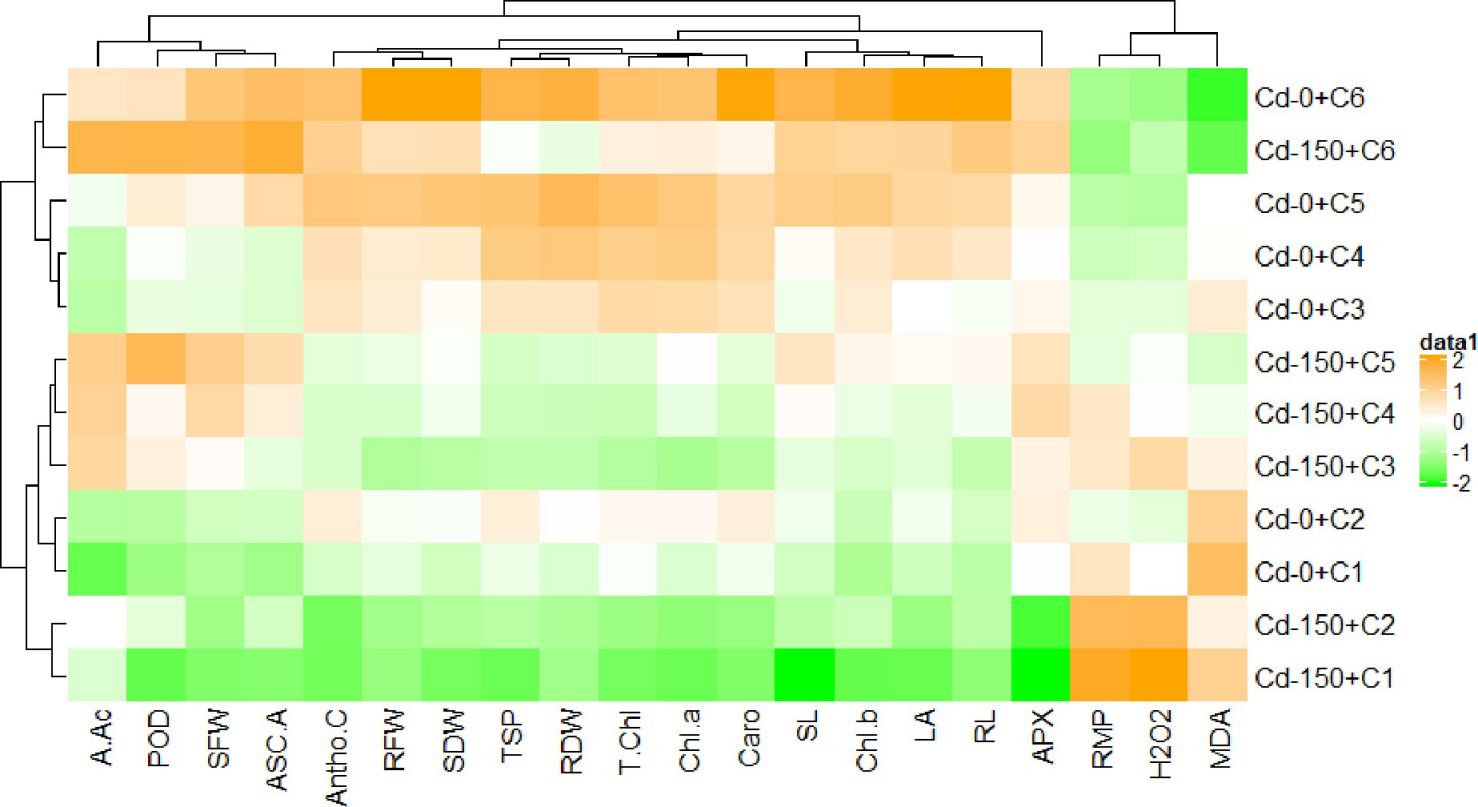
Clustered heatmap representing the effect of Cd and Ca (C1- 0 m*M*, C2- 0.5 m*M*, C3- 1 m*M*, C4- 2.5 m*M*, C5- 5 m*M*, C6- 10 m*M*) treatments on different studied traits. Abbreviations are given at start of manuscript.

A clustered heatmap was constructed to evaluate the effect of Cd and Ca treatments on the different traits as shown in Fig 5. Under higher concentrations of Ca (10 mM), RMP, H_2_O_2_ and MDA showed a significant reduction in response to 0 and 150 μM concentrations of Cd indicating a parallel response in both treatments. A noteworthy influence of 10 mM level of Ca in non-stressed seedlings (0 μM Cd) was recorded with a greater increase in growth traits (RFW, RDW, SFW, SDW, SL, RL, LA), chlorophyll (Chl *a* & *b*, T. Chl), organic osmolytes (TSP), anthocyanin contents (Antho. C) and ascorbic acid (ASC.A). All these traits were tightly grouped together and indicated high performance of 10 mM level of Ca under non-stressed conditions. In Cd stressed (150 μM) seedlings, 10 mM level of Ca contributed to a significant increase in amino acids (A.AC), peroxidase (POD), ascorbic acid (ASC.A) and shoot fresh weight (SFW). Shoot length (SL), root length (RL), leaf area (LA), the activity of ascorbate peroxidase (APX) and chlorophyll showed a strong and clear similarity and strongly clustered together. Antioxidants (APX and POD), ascorbic acid (ASC.A), anthocyanin contents (Antho. C) reduce the RMP, H_2_O_2_ and MDA and are clustered together in the same group. At the highest level of Ca (10 mM), clustering and similarity indicated a high performance and a possible relationship between different traits under stress treatments.

### Response of different traits under stressed and non-stressed conditions

**Fig 6:**
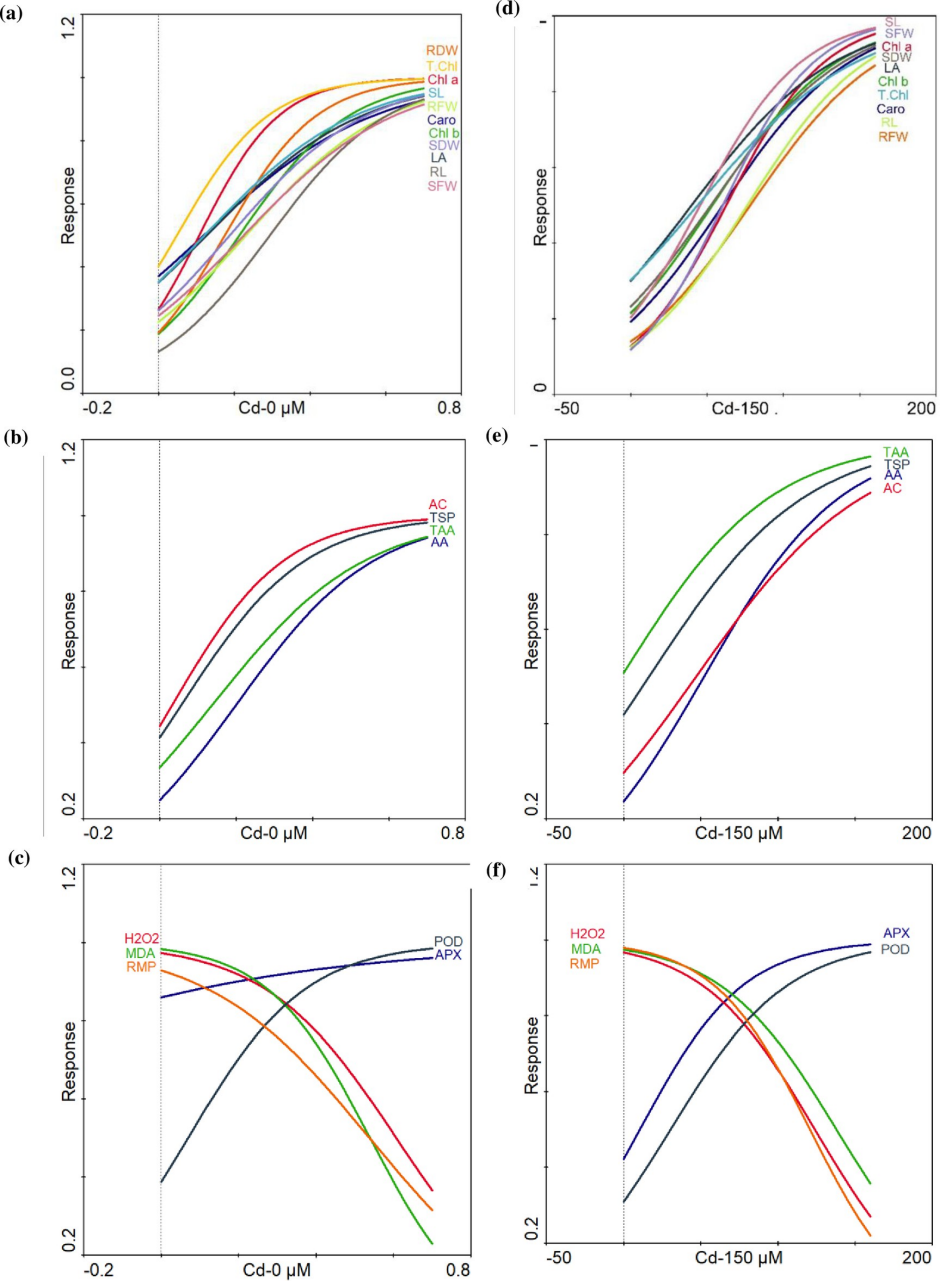
Generalized linear model showing response curve of traits under Cd and Ca treatments. **Cd- 0 μ*M* stress: (a)** growth and chlorophyll **(b)** organic osmolytes, ascorbic acid and anthocyanin contents **(c)** hydrogen peroxide, relative membrane permeability, lipid peroxidation and antioxidants. **Cd- 150 μ*M* stress; (d)** growth and chlorophyll **(e)** organic osmolytes, ascorbic acid and anthocyanin contents **(f)** hydrogen peroxide, relative membrane permeability, lipid peroxidation and antioxidants. Abbreviations are given at start of manuscript.

In non-stressed conditions (0 μM), a conspicuous positive response was observed for the growth traits (RL, SL, SFW, SDW, and LA) and chlorophyll (Chl *a*, Chl *b* and T. Chl) as Ca levels increased (Fig 6A). Organic osmolytes (TAA, TSP), anthocyanin contents (AC) and ascorbic acid (ASc-A) showed a sharp positive response with increasing Ca regimes (Fig 6B). H_2_O_2_, MDA and RMP exhibit a strong negative response with an increase in Ca levels, however, APX and POD exhibit an increasing pattern in curve with elevated Ca gradients (Fig 6C). In Cd stressed conditions (150 μM), growth traits (RL, SL, SFW, and SDW, LA) and chlorophyll (Chl *a*, Chl *b* and T.Chl) displayed a strong positive response and in response to Ca levels (Fig 6D). The concentration of TAA, TSP, AC and TAA was the maximum with a positive response (Fig 6E). A strong positive response was noted in the activity of APX and POD along with increasing Ca levels. In contrast, a strong negative response was assessed for H_2_O_2_, MDA and RMP with an increase in Ca regimes (Fig 6F).

## Discussion

Calcium plays an essential role in the mitigation of abiotic stresses and protection from drastic impacts [24, 48–50]. It interacts with proteins like calmodulin to up-regulate gene expression and regulate the movement of metal ions across membranes [51]. The present work demonstrated that Ca significantly alleviated the toxic effect of Cd in maize by improving all growth traits. Furthermore, the alleviation of Cd toxicity was more obvious at higher treatment levels of Ca in stressed and non-stress plants. Previous studies revealed that Ca applications regulate the uptake of heavy metal ions as it competes for transporter sites on plasma membrane [24]. Supplemented Ca^2+^ reduced Cd toxicity by enhancing growth traits as reported in other crops like in mustard [24] and rice [52]. Additionally, Ca reduced the toxic effect of nickel in rice seedlings [53] Calcium is essential for plants and is involved in the various physiological processes, cell division, and photosynthesis and interacts with intracellular signal transduction. Due to chemical similarity with Cd, Ca mediate various Cd-mediated physiological and metabolic processes [24]. Recent works elaborated that Ca use as an exogenous supplement to prevent the noxious impacts of the Cd [28].

Reduction in growth traits under Cd toxicity is directly linked to the reduction of photosynthetic contents. As anticipated, the photosynthetic pigments significantly declined under 150 μM Cd treatment level. However, higher levels of Ca significantly improved carotenoids, total Chlorophyll (Chl), Chl *a*, *b* pigments in maize seedlings under Cd stress (Table 2). Previously, the interactive effect between Ca and heavy metal was reported in some studies where exogenously applied Ca significantly prevented the damaging effects of Cd on photosynthetic pigments [53, 54]. Calcium is also obligatory required to activate the oxidation of H_2_O oxidation, maintain photochemical efficiency of PSII, and restore photosynthesis by aggravating the concentrations of the photosynthetic pigments [55, 56]. Calcium is a divalent cation and shares many parallel physical properties (like pH) with divalent heavy metals like Cd, Ni, and Co [57]. Therefore, exogenously applied Ca ions through the rooting medium can successfully restrict the uptake of Cd metal ions through competition for uptake and transport in plants [57]. In current work, the enhanced amount of photosynthetic pigments in Cd treated maize seedlings seemed to be a direct effect of enhanced activities of anti-oxidative enzymes, and other protective molecules that reduced membrane damage [58].

The improvement in the antioxidant defense system enables plants to alleviate heavy metals toxicity [53]. In the current study, APX and POD activities significantly improved in Ca treated plants under Cd toxicity. These results suggest that applied Ca effectively alleviate Cd-induced oxidative stress [59]. Under heavy metal stress, Ca activates diverse protein kinases and strengthens the antioxidant defense system [20, 60]. Tolerant plants had evolved an efficient antioxidant system to balance the concentration of reactive oxygen species [28, 61]. Enzymes like APX and POD also take part in the detoxification of free radicles and lead to sequestering of H_2_O_2_ [62]. APX is mainly localized in chloroplast, apoplast, cytosol, mitochondria, and peroxisome and POD in cell walls, cytosol, and vacuoles, Both APX and POD are mainly implicated to scavenging the H_2_O_2_ [61]. Their efficiency is enhanced during Cd stresses and that greatly imparts stress tolerance and modulates the physiological process in maize seedlings in this study [63, 64]. Exogenous application of Ca remarkably decreases the intracellular level of Cd by activation of antioxidant defense mechanism to a level capable of suppressing the generation of ROS. This suppression is corroborated by the enhancement of APX, SOD, POD and CAT to efficiently scavenge the toxic ROS [33]. Activation of these antioxidants attributed to photosynthetic efficiency and perception of stress signals by elevation of cytosolic Ca as an early signal event, known as Ca signature. This Ca signature is detected by Ca sensors and then a downstream signal is transduced that subsequently enhances the defense mechanisms [65].

Plants exposed to metal stress showed alterations in cell membrane permeability (RMP) and consequently, the cell loses membranes integrity [66]. Cell membrane integrity is considered as a tool to regulate ionic movements and use as a selection criterion to quantify damage magnitude. In current results, the relative RMP markedly increased under Cd stress. However, the RMP significantly was markedly reduced by the Ca treatments that alleviated the damaging consequences of Cd. In plants exposed to Cd stress, relative membrane permeability (RMP) substantially increased and caused membrane impairments [67]. Under Cd stress, supplemented Ca decrease the electrolyte leakage that showing the defensive role of Ca to increase the membrane stability [68]. Calcium mainly stabilizes the membrane integrity and also controls the movement of divalent cations and prevent solute leakage by reducing peroxidation of lipids [52, 69]. In addition, Ca is known to maintain the membrane integrity that is concomitant with Ca-chelators to target the ROS and reduce the lipid peroxidation [70].

The scavenging of excessive ROS is a vital process by regulates the regular operation of a cellular system. Excessive ROS can be rid of by inbuilt antioxidants defense system attributed to enzymes viz, MDHAR, DHAR, GR, APX, POX, CAT, SOD and non-enzymes DHA, AsA, GSSG and GSH [33].

The excessive accumulation of both MDA and H_2_O_2_ under metal stresses damages biomolecules by excessive lipid peroxidation, degrades membranes, decreases photosynthesis and hampered the activity of other essential enzymes [61]. Plants enhance the antioxidant system to deplete the ROS which ultimately reduces oxidative stress generated by high metal concentrations [71]. The Ca applications as observed in this study, improved the activities of various antioxidants (enzymatic or non-enzymatic) and reduced the level of H_2_O_2_ and lipid peroxidation [20, 72]. Previous studies authenticate the pivotal role of Ca to prevent the accumulation of cellular Cd and improve ROS-scavenging capacities that led to the reduction of ROS in plants [33]. Calcium also up-regulates genes that are responsible to encode the antioxidant under oxidative stress [73]. In the present work, the level of ROS increased under Cd stress, however, the addition of Ca considerably reduced the production of ROS in maize seedlings (Fig 1).

Anthocyanin belongs to flavonoids and naturally occurs in water-soluble plant pigments [74]. In plants, anthocyanin plays a pivotal physiological role as scavenges free radicles, increases the organic osmolytes and photosynthetic efficiency [75, 76]. The biosynthesis of anthocyanin is regulated by environmental and developmental signals [77, 78]. The excessive accumulation of anthocyanin is regarded as defense mechanism [79]. The anthocyanin contents remarkably increased in the present study which was more pronounced in the highest levels of Ca (Fig 1) that is are parallel to many previous findings [60, 80]. A high level of anthocyanin regulates heavy metal transport toward the vacuole and sequestration [81]. Exogenously applied calcium is reported to reduce Cd toxicity by stimulating the synthesis of glutathione-S-transferase (GST) enzyme to increase anthocyanin contents that in turn ameliorates the oxidative stress by scavenging the free radicals [81].

Heavy metal stress causes determinal changes in cellular structures and causes osmotic stress [82]. Plants mitigate osmotic stress by accumulating the lower or higher weight osmolytes that do not hinder the functioning of important metabolites [83]. During Cd stress, plants employed several protective strategies to reduce the noxious impacts of Cd stress [17]. Osmolytes primarily reduce water potential and ensure the water balance [84], protects subcellular structures, and reduce oxidative damage [85]. Plants accumulate the organic osmotica to maintain the tissue water contents and upregulate the working capabilities of antioxidants during stressful conditions [86, 87]. Amino acids act as organic osmolytes and participate in osmotic adjustments, stabilize proteins in membranes [88], ion homeostasis [47], scavenges the ROS and neutralize the redox potential during oxidative stress caused by noxious heavy metals [88]. Calcium is an indispensable element for plant osmotic adjustments and increase the levels of free amino acids under heavy metal stresses [89]. In the present studies, the seedlings showed more accumulation of osmolytes under the application of Ca (Fig 2). Ascorbic acids (ASc) are non-enzymatic antioxidant enzymes, which act as a cofactor for many important enzymes and accumulate in leaves [63]. Ascorbic acid plays a crucial role in protecting the cellular metabolism from oxidative damage by acting as a reductant [90]. It serves a defensive role during oxidative stress and reduces the H_2_O_2_ and detoxifies the free radicals [91]. In the present study, ascorbic acid in maize seedlings was significantly enhanced by the addition of Ca (Fig 2). Foliar applications of AsA potentially alleviate the Cd toxicity in maize by modulating the physio-chemical attributes, boosting the activities of antioxidant enzymes, and improving the photosynthetic process, and concentrations of organic osmolytes [92].

Calcium, a ubiquitous messenger, is well known to regulate metabolic processes, act as a transducer, regulate photosynthesis and balanced the level of essential nutrients [93]. A slight change in an intracellular Ca concentration can modulate a large array of fundamental biological processes such as growth, physiology and biochemical process under heavy metal stress [94]. In the present work, Ca modulated various fundamental processes such as growth, physiological and biochemical processes in maize seedlings which ultimately enhanced Cd tolerance. Supplementation of Ca to plants actively participates in heavy metals tolerance mechanisms [95]. Exogenously applied Ca^2+^ enhanced activities of cellular antioxidants such as APX, POD which were helpful to restrict the production of ROS to prevent oxidative damage [24]. Several studies confirm the importance of the Ca^2+^ by induction of Cd tolerance since physio-biochemical characters of Ca^2+^ are quite similar to that of Cd^2+^, these similarities result in the replacement of Cd^2+^ with Ca^2+^ metal. Thus, Ca^2+^ uptake by receptors/ channels can be enhanced which increases Ca^2+^ storage viability in plants under Cd stress because of the similarity in ionic radii of both metals [19, 96]. Calcium (Ca) also works as a second messenger in plants, which underpins the abiotic stress-induced damage. However, the sequence of action of these signalling molecules against cadmium (Cd)-induced cellular oxidative damage remains unrevealed [33]. In this prospect, more work can be done to understand the exact mechanism of the Ca induced mitigation of Cd stress by enhancing the growth, physiochemical and genetic approaches.

## Conclusions

In conclusion, Cd-induced oxidative stress caused negative influences on the growth and physio-biochemical traits of plants. In response to Cd stress, plants got triggered their defense mechanisms, nonetheless at the same time, Cd stress increased the level of stress markers (MDA, H2O2) and plants are unable to handle Cd-induced cellular impairment as witnessed by elevation in RMP and reduction in anthocyanin contents (ACC). Plants showed a low level of organic osmolytes (TSP, Amino acids) under Cd stress. Exposure to Cd caused a reduction in growth traits (SL, RL, SFW, SDW, RFW, RDW), a reduction in activities of antioxidants (APX, POD), and markedly declined the photosynthetic pigments (Chl a, Chl b and T.Chl). However, supplementation of Ca markedly reduced the oxidative stress by elevating the level of antioxidants (APX, POD), and non-antioxidants (ascorbic acid) to scavenge the ROS. Exogenously applied Ca ameliorated the oxidative stress by increasing the level of organic osmolytes as total soluble proteins, and free amino acids to maintain the integrity of cellular membranes and cell osmotica. Maintenance of organic osmotica causes an increase in antioxidant enzymes that ultimately suppressed ROS and enhanced the accumulation of organic osmolytes. Collective responses are reflected in the form of improved growth and more photosynthetic pigments. Therefore, Ca supplementation under Cd stress, enabled the maize to counter determinal effects of Cd-induced damages. In future prospect, the biosynthetic pathways involved in the up-regulation of antioxidants and organic osmolytes should be further investigated.

## Abbreviations

MDA: Malondialdehyde
RMP: Relative membrane permeability
H_2_O_2_: Hydrogen peroxide
RFW: Root fresh weight
RL: Root length
T. Chl: Total chlorophyll
SDW: Shoot dry weight
ASC.A: Ascorbic acid
Caro: Carotenoids
Antho-C, ACC: Anthocyanin contents
Chl *b*: Chlorophyll *b*
Chl *a*: Chlorophyll *a*
TSP: Total soluble proteins
LA: Leaf area
RDW: Root dry weight
POD: Peroxidase
SFW: Shoot fresh weight
APX: Ascorbate peroxidase
A-Ac: Amino Acids
SPB: Sodium phosphate buffer
